# Dark Topics on Giant Retroperitoneal Liposarcoma: A Systematic Review of 157 Cases

**DOI:** 10.3390/cancers17050740

**Published:** 2025-02-21

**Authors:** Alfonso Santangelo, Agostino Fernicola, Domenico Santangelo, Gaia Peluso, Armando Calogero, Felice Crocetto, Akbar Jamshidi, Luigi Pelosio, Alessandro Scotti, Vincenzo Tammaro, Valentina Tranquillo, Dario Tammaro, Carmen De Cocinis, Francesca Della Gaggia, Emanuela Capezio, Nicola Carlomagno, Michele Santangelo

**Affiliations:** 1 Unit of Urology, Division of Oncology, Gianfranco Soldera Prostate Cancer Lab, IRCCS San Raffaele Scientific Institute, Vita-Salute San Raffaele University, 20132 Milan, Italy; santangelo.alfonso@hsr.it; 2 Department of Advanced Biomedical Sciences, Unit of Emergency Surgery, Federico II University, 80131 Naples, Italy; armando.calogero2@unina.it (A.C.); akbar.jamshidi@unina.it (A.J.); luigi.pelosio@unina.it (L.P.); alessandro.scotti@unina.it (A.S.); vincenzo.tammaro@unina.it (V.T.); nicola.carlomagno0804@gmail.com (N.C.); misantan@unina.it (M.S.); 3 Department of Radiology, Vita-Salute San Raffaele University, IRCCS San Raffaele, 20132 Milan, Italy; santangelo.domenico@hsr.it; 4 Surgical and Biomorphological Sciences PhD, Federico II University, 80138 Naples, Italy; 5 Department of General Surgery, Fatebenefratelli Hospital of Naples, 80123 Naples, Italy; gaia.peluso5@gmail.com; 6 Department of Neurosciences, Sciences of Reproduction and Odontostomatology, University of Naples Federico II, 80131 Naples, Italy; felice.crocetto@unina.it

**Keywords:** giant retroperitoneal liposarcoma (giant RPL), giant retroperitoneal liposarcomas (giant RPLs), huge retroperitoneal liposarcoma, enormous retroperitoneal liposarcoma, giant liposarcoma of the retroperitoneum, huge liposarcoma of the retroperitoneum, massive liposarcoma of the retroperitoneum, enormous liposarcoma of the retroperitoneum

## Abstract

Background/Objectives: Giant Retroperitoneal Liposarcomas (giant RPLs) represent a rare malignant disease of adulthood that does not yet have a univocal definition in the scientific literature. The symptoms may be late, depending on the position and the size reached. The weight may exceed 20 kg, and the diameter 25 cm. The main treatment is the surgical approach. This systematic review aims to collect data from the present literature and to answer some questions on the nature of this pathology. Methods: We performed a search on the PubMed, Cochrane, and Scopus databases using specific search strings. Non-English written articles and abstracts were excluded. Results: Dimensional, histological, and pathological data of giant RPLs were extracted and recorded in an electronic database, and charts were used to synthesize the results. We selected 126 manuscripts, all case reports and case series, and obtained data for 157 giant RPLs. The major axis varied from 15 to 80 cm, and the weight ranged between 2.5 and 98 kg. Sex distribution was homogenous. Age was reported 146 times, and almost 1/3 of the study population was under 50 years old. The most frequent hystotype reported was well-differentiated liposarcomas, while the rarest was pleomorphic liposarcomas. In 139 cases, the symptoms were reported and generally included a mass effect on surrounding organs. The exclusive surgical operation was the most frequent treatment option, and it included both the resection of the tumor and other organs involved. Chemo- and radiotherapy were also performed, in a few cases. In 36 reports, distant metastasis was suspected, but only 6 were effectively positive. Conclusions: Giant RPLs are a clinical entity that shares some common features with normal-size liposarcomas but are different in dimensions, age distribution, histologic prevalence, rate of incidental diagnosis, organ sparing, and R0 resection. More studies are needed to completely characterize these tumors.

## Simple Summary

This systematic review analyzes and compares a large number of cases of a rare adult malignant tumor: the giant retroperitoneal liposarcoma (giant RPLs). In the literature, the data reported by the different authors in terms of definition, diagnosis, and treatment are not univocal and uniform. This can lead to a certain disambiguity in the correct classification of this pathology. We focused our attention on a total of 157 cases of retroperitoneal liposarcomas defined as “giant” and analyzed homologies and differences. Therefore, this review aims to clarify these gaps and controversies present in the scientific literature and thus improve the global understanding of this rare tumor. The results indicate that further studies are needed to better define this cancer and thus improve the diagnostic and therapeutic approach.

## 1. Introduction

Retroperitoneal sarcomas (RPSs) are rare malignant tumors that represent 15% of all sarcomas with an overall incidence of 0.3–0.4% per 100,000 members of the population [1]. Retroperitoneal liposarcomas (RPLs) are sarcomas arising from the retroperitoneal fatty tissues that alone account for 41% of all RPSs and for 0.07–0.2% of all tumors [2].

RPLs are categorized into five major subtypes: well-differentiated liposarcomas (WDLPSs), which are subcategorized into sclerotic, inflammatory, and lipomatous forms; dedifferentiated liposarcomas (DDLPSs), which can also develop from a former WDLPS; myxoid liposarcomas (MLPSs); pleomorphic liposarcomas (PLPSs) and mixed forms, the last having a more aggressive clinical course [2,3].

Every histological subtype of RPLs has a direct correlation with a specific anatomic site: WDLPSs and DDLPSs arise preferentially in the retroperitoneal cavity, while MLPSs and PLPSs arise in the extremities [3,4]. The gold standard treatment for RPL is an R0 resection because their prognosis depends on the radicality of the surgical resection [5]. Unfortunately, the risk of recurrence is high even when a radical excision is performed [5].

The retroperitoneal cavity represents an optimal site for tumor expansion since a mass can enormously grow before compressing another organ and hence become symptomatic. Therefore, RPLs can present large dimensions at the time of the diagnosis (20–25 cm) and, in some cases, can also be defined as giant or huge [3].

Although giant RPLs are not that rare, specific articles dedicated to them are very few. Therefore, for their characterization, reference is made to the information acquired from smaller RPLs.

In fact, when analyzing the literature, we found a scarcity of reviews. Furthermore, the information on giant RPLs comes mainly from occasional case reports or case series. Due to this lack of information on RPL giants and the confusion surrounding this topic, we decided to review the scientific literature and collect data on each reported case.

## 2. Materials and Methods

We performed a systematic review on PubMed, Web of Science, Scopus, and EMBASE from 1 September 2004 to 31 May 2023 using the following keywords “giant retroperitoneal liposarcoma” AND “huge retroperitoneal liposarcoma” OR “enormous retroperitoneal liposarcoma” AND “giant liposarcoma of the retroperitoneum” OR “huge liposarcoma of the retroperitoneum” OR “massive liposarcoma of the retroperitoneum” AND “enormous liposarcoma of the retroperitoneum” to find multiple records of this malignancy.

The types of articles included were Care Reports and Case Series. We excluded manuscripts written in languages other than English and for which only the abstract was available. Incomplete and non-peer-reviewed preprint articles were excluded. All articles had to have a retroperitoneal liposarcoma defined as giant or huge as the primary pathology treated. We included articles that addressed giant RPLs both surgically and pharmacologically and articles that reported resection margin status and outcomes for R0 and R1 resections. In accordance with the residual tumor classification (*R classification*) guidelines established by the *American Joint Committee on Cancer* (AJCC), an R0 resection was defined as the absence of tumor cells on the inked resection surface, and an R1 resection was defined as microscopic tumor cells present at the edge of the specimen, whereas an R2 resection indicates the presence of a macroscopic residue of disease (i.e., a portion of the tumor visible to the naked eye).

The abstract of each article was screened by three authors and the selected articles were evaluated in full text by three other authors. Eligible articles received approval from at least two of the three authors who performed the full-text screening. After full-text evaluation, all articles were included according to their relevance according to the topics of the article: definition, age and sex distribution, therapeutic approaches, and incidence of local recurrences and metastasis of giant RPLs. Furthermore, we examined the full-text reference lists of the evaluated articles to identify any other relevant articles, and these articles were evaluated according to the same criteria. We have not registered this research in a public registry such as PROSPERO or the Open Science Framework or Inplasy.

The aim of our review was to answer the following questions:

- What is the correct definition of giant RPLs?
- Are giant RPLs equally distributed across age and sex?
- What is the most common histology of giant RPLs?
- How often is it diagnosed incidentally?
- How often is surgery associated with R0 resections and how often is it used for palliation?
- How often are radiotherapy and chemotherapy included in the treatment regimen?
- What is the incidence of local recurrence and metastases?
- What is the possibility of sparing abdominal organs, and which are the most common organs removed with the tumor?

To answer these questions, we collected the following data for all giant RPLs identified by the selected articles:

- Age and sex of each patient.
- Symptoms associated with giant RPL.
- Dimensions (cm) of giant retroperitoneal liposarcoma (giant RPL).
- Weight (kg) and histotype of the excised specimen.
- Aim and result of the therapy.
- Follow-up timing.
- Occurring of local recurrence/metastasis.
- Organs involved in the surgical excision.

For data explanation, we created charts reporting the number of citations of every article; however, when an article contained information about more than one tumor a dot was used to clarify the data (i.e., number 19.2 refers to the second giant RPL reported in the article 19).

## 3. Results

**Figure 1:**
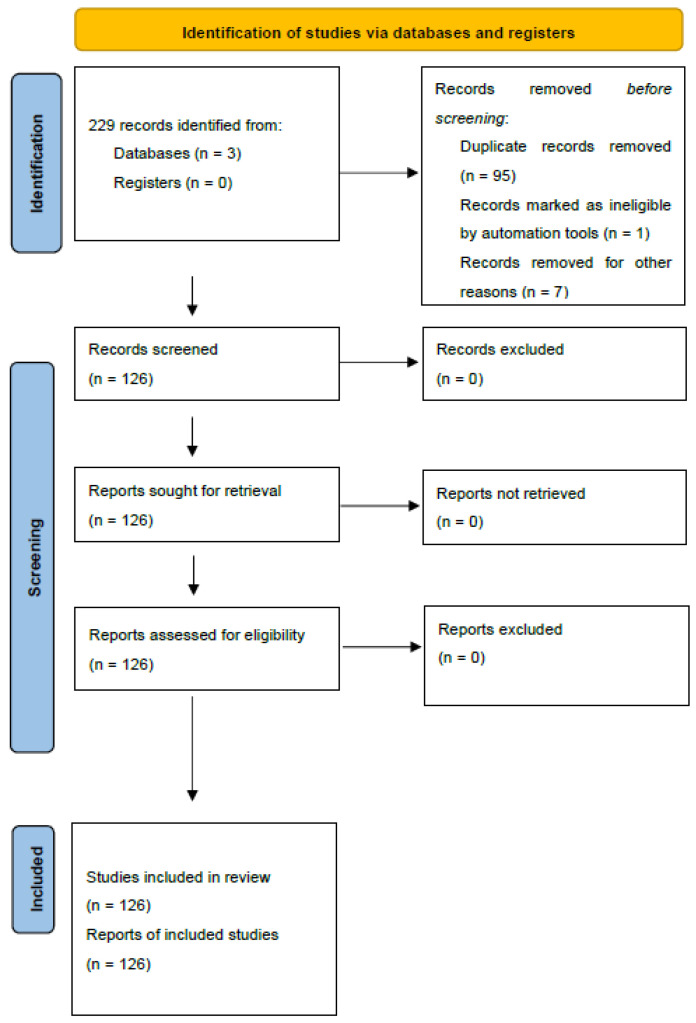
Selection process: PRISMA 2020 flow diagram for reviews.

We first selected 134 articles; 8 of them were excluded because the full text was not available or was written in a language other than English. Of the remnant manuscripts, we obtained data for 157 giant RPLs [6,7,8,9,10,11,12,13,14,15,16,17,18,19,20,21,22,23,24,25,26,27,28,29,30,31,32,33,34,35,36,37,38,39,40,41,42,43,44,45,46,47,48,49,50,51,52,53,54,55,56,57,58,59,60,61,62,63,64,65,66,67,68,69,70,71,72,73,74,75,76,77,78,79,80,81,82,83,84,85,86,87,88,89,90,91,92,93,94,95,96,97,98,99,100,101,102,103,104,105,106,107,108,109,110,111,112,113,114,115,116,117,118,119,120,121,122,123,124,125,126,127,128,129,130,131,132,133,134,135,136,137,138,139]. The selection process and the number of cases for each paper are explained in Figure 1.

### 3.1. Dimensions of Giant RPLs

**Figure 2:**
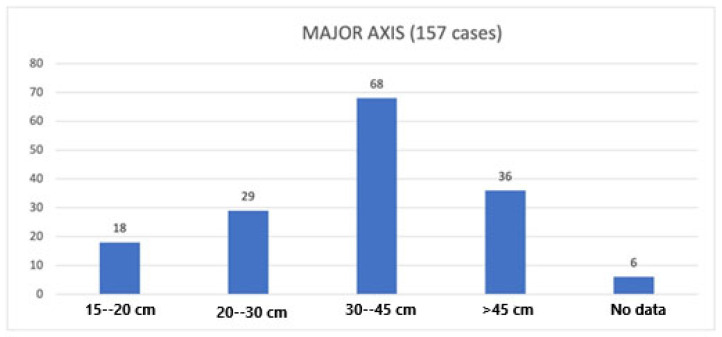
Data about dimensions [Abscissa (X): length of the major axis; Ordinate (Y): number of giant RPLs].

We considered the major axis of each tumor to assess tumor size. The minimum diameter was 15/16 cm, reported by Huo et al. [49], and the maximum was 80 cm, reported by Herrera-Gomez et al. [24]. In two cases, the length was obtained by the sum of the different masses composing the giant RPLs [2,134]. We reported the data regarding the major axis in Figure 2.

### 3.2. Weight of Giant RPLs

**Figure 3:**
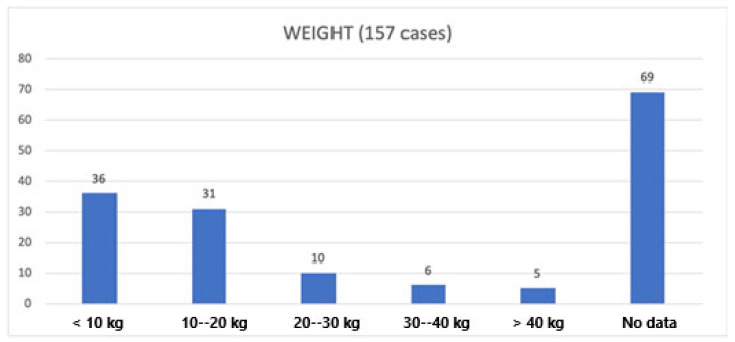
Data about weight.

The weight of the excised specimen was reported only in 88/157 cases (Figure 3). The described weights ranged between 2.5 kg and 98 kg.

### 3.3. Patients’ Sex

Patients’ sex was mentioned in 156 cases, with 82 males and 65 females, and it was not reported in only 1 case [22]. In 9 cases, the giant RPLs were relapses that occurred in patients already counted in this study.

### 3.4. Patients’ Age

**Figure 4:**
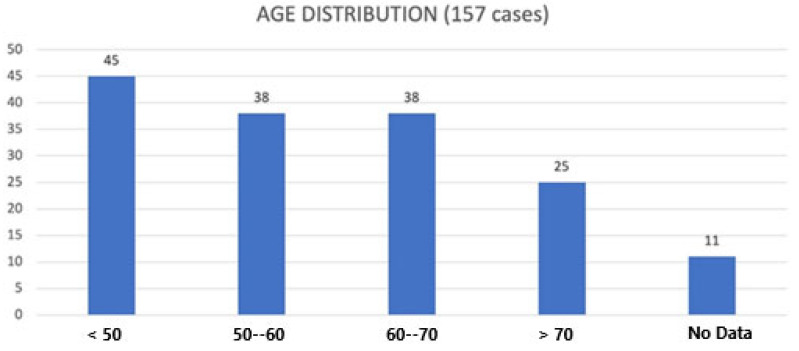
Data about patients’ age distribution [Abscissa (X): patients’age; Ordinate (Y): number of patients with giant RPLs].

Patients’ age was reported in 146 cases (Figure 4). In the 9 patients with giant relapses, the age was considered both at the time of the first diagnosis and at the time of the recurrences.

### 3.5. Histology of Giant RPLs

**Figure 5:**
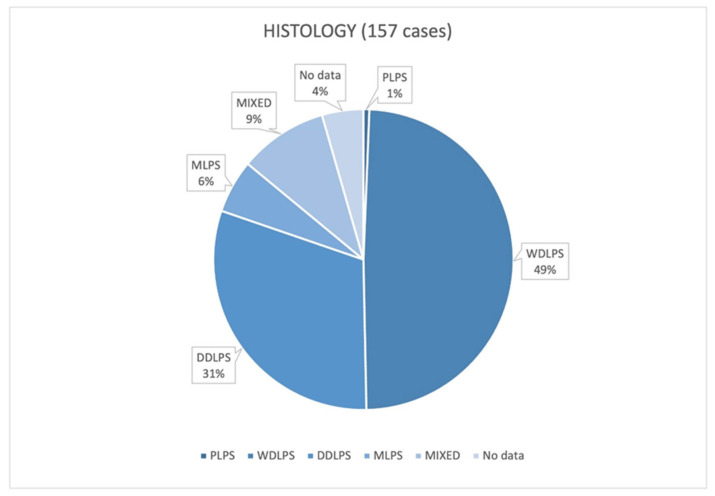
Data about histology [Well differentiated liposarcomas (WDLPSs), Dedifferentiated liposarcomas (DDLPSs), Myxoid liposarcomas (MLPSs), Pleomorphic liposarcomas (PLPSs)].

These data were reported in 150 cases (Figure 5).

### 3.6. Clinical Presentation

**Figure 6:**
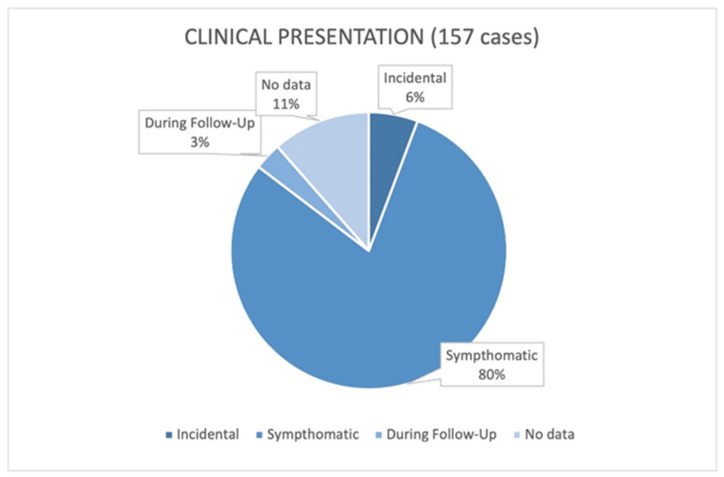
Clinical presentation.

A total of 125 patients had palpable and symptomatic masses at diagnosis: the main ones were weight gain, abdominal distension, and mass effect on surrounding organs; 14 patients were asymptomatic at diagnosis, and 9 patients were incidental, 3 of which were found in pregnant women during routine examinations [49,66,94], and 6 were totally incidental; 5 cases were recurrences (major axis: >15 cm) found during follow-up (Figure 6). For the other 8 patients, there was no information on clinical features [6,125,128].

### 3.7. Treatment

**Figure 7:**
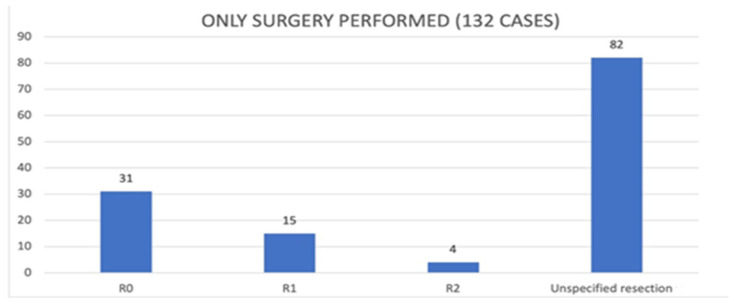
Data regarding surgery [Abscissa (X): residual tumor classification (R classification); Ordinate (Y): number of patients with giant RPLs].

Regarding surgery, data were reported in 156 out of 157 giant RPLs; in one case, data were not reported [27]. A total of 153/156 cases underwent a surgical operation, while, in 3/156 cases, surgery was not performed. The exclusive surgical approach was used in 132 cases; of these, in only 50 cases were the resection margins expressly declared (Figure 7).

Chemotherapy and radiotherapy were also carried out. Postoperative radiotherapy was performed in 10 cases: 2 after an R0 resection [39,72]; 1 after an R1 resection [47]; 7 after an unspecified resection [6,30,67,83,92,124,125].

Postoperative chemotherapy was administered in 7 patients: 2 after an R0 resection [127]; 1 after an R1 resection [136]; 1 after an R2 resection [41]; 3 after an unspecified resection [93,94,125].

Postoperative radio-chemotherapy association was reported twice: 1 after an R0 and 1 after an R1 resection [95,99].

Neoadjuvant radiotherapy was reported only twice by Ostrominski et al. and Cheng et al. and, in both cases, it was followed by an R2 resection margin [7,119]. In particular, the therapeutic strategy adopted by Cheng et al. included an adjuvant RT followed by an R2 resection, 6 cycles of CT, another R2 resection, and a new CT protocol [7].

### 3.8. Local Recurrence and Metastasis

Regarding local recurrence, data were reported in 96/157 cases, and, in the other 61/157, recurrence data were not specified. Local recurrence occurred in 33/96 cases. The time of recurrence was specified only 10 times: 3 recurrences presented within 12 months [30,50,83]; 7 cases between 12–34 months [80,84,86,125,133]. A total of 2 giant RPLs had 2 recurrences [28,32]; only one giant RPL had 3 recurrences [56].

Distant metastases were cited in 36 articles, while, in 121/157 cases, no data were reported. In 30 cases, metastases were expressly declared as absent. Of the remaining 6 cases, 3 cases were found in DDLPS, 2 cases in mixed RPLs, and 1 case in MPLS [17, 50, 86, 128.4, 128.5, 128.6].

### 3.9. Organs Involved in the Surgical Excision

The resection of the giant RPLs often included the other organs’ exeresis. Regarding this topic, we considered only 147 cases because the 10 cases reported by Bachman et al. gave us only cumulative information [128].

**Figure 8:**
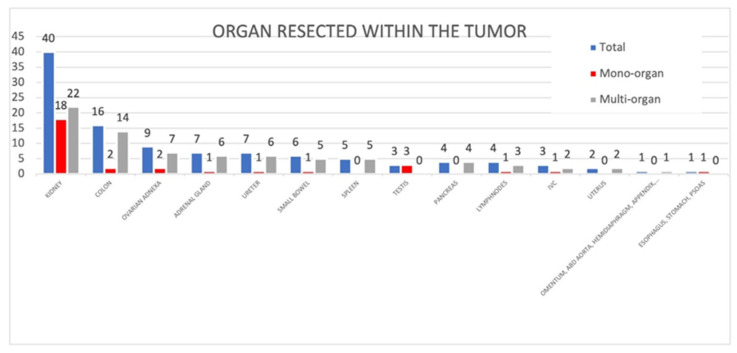
Data about organ excision [Abscissa (X): type of organ resected within the giant RPLs; Ordinate (Y): number of patients with giant RPLs].

Out of these 147, specific organ resection was declared 66 times, while organ sparing was declared 35 times. In the other 46 cases, there was no explicit reference to this topic. Kidneys were the most resected organs, followed by the colon in any tract. Mono-organ resection occurred in 31 cases while multi-organ excision happened 35 times. The organs resected are specified in Figure 8.

Regarding data reported by Bachman et al. and not included in Figure 8, resection included 4 kidneys, 2 colons, 2 adrenal glands, and 2 testes in 10 total cases [128].

## 4. Discussion

Our results confirm that some features of giant RPLs are not shared among all the authors. Anyway, the results of this review answer some previous questions and form the basis for further characterization of giant RPLs. When analyzing the literature, we found few reviews and no controlled studies on this topic, and, globally, the information on giant RPLs comes mainly from occasional case reports or limited case series. The only study with a considerable number of patients was published by Deng et al. [5]. This is a monocentric retrospective study of 61 cases treated between 2000 and 2015 [5]. For this reason, considering the characteristics of the population (same nationality/ethnicity/lifestyle habits), we decided to take this study as a reference to compare our data when possible. This study has limitations. In fact, it included only articles published in English, thus excluding publications in other languages. Furthermore, this study did not assess the quality of the included studies. The results do not provide specific data, such as pain scores, patient satisfaction after the therapeutic procedure, and post-operative complications, because not all authors reported them. A future move towards prospective research is essential to better understand the aspects of giant RPL in the oncological landscape.

### 4.1. What Is the Real Definition of Giant or Huge RPLs?

In the literature, there is much confusion when defining a giant/huge/enormous RPL, and every author has his own dimensional cut-off. Thus, some authors define giant RPL tumors as tumors whose major axis is less than 20 cm [8,19,40,49,58,71,80,83,95,96,125,129,130,136,137]. This same size is considered normal by others [2,3]. Instead, some authors consider a cut-off of between 20 and 30 cm [140,141]; for other authors, the cut-off is greater than 40 cm [36]. Finally, Bachman et al. argue that, to define a giant RPS, it is necessary to find a tumor with a long diameter that is twice as large as the length specified for the pT4 category (≥30 cm) [5,123]. This nosographic confusion has prompted us to suggest a more specific classification. In our opinion, the term Big RPL can be used to describe tumors of between 20 and 30 cm. In fact, these represent 18% of our cases (29/157). We suggest that a more adequate definition of Giant RPL should be reserved for tumors between 30 and 45 cm, which represent 43% of the cases examined (68/157), while, for tumors >45 cm (22% (36/157) of the tumors in our study) an adequate term could be Huge RPL or Enormous RPL.

To define an RPL as big/giant/huge, some authors tend to also consider the weight of the excised specimen, with a cut-off of around 20 kg [3,39,141]. This parameter has been reported by 83 authors, varying from 2.5 kg to a value of 98 kg [55]. We believe that the weight of the tumor cannot be considered a parameter to define a giant RPL because liposarcomas are mainly made of fat, which is lighter than water and is, therefore, lighter than any other invaded tissue. It is obvious that greater weights will also depend on the excised organs within the tumor. Another key factor to consider is the histology: WDLPs are made almost entirely of fat while other histotypes are made of different tissues; therefore, tumors of the same size will have a different weight depending on their histology.

### 4.2. Do Giant RPLs Equally Distribute Between Age and Sex?

The distribution of giant RPLs in our study was almost equal between the sexes, thus suggesting that it does not represent a risk factor for this neoplasm.

These data are in line with both Deng et al. and the literature on normal-sized RPS and RPLs, neither of which show any difference in racial populations [1,2,5].

On the other hand, normal RPLs are mainly observed in an age range between 50 and 70 years [2]. However, through our review, we found that 31% of patients were younger than 50 years. Specifically, 45 patients are part of this group. Half of them were <40 years old and the other were half between 40 and 50 years. The hypotheses for this distribution may be various (e.g., hormonal causes). However, given the small number of patients in this group, we believe that no correlation with sex can be made. Therefore, for a possible correlation between age (younger than 50 years) and sex (male and female), larger patient samples are needed in the future.

Our results were substantially consistent with those of Deng et al., who reported slightly higher ages with ages ranging from 46 to 63 years [5]. This last data could be related to the probable ethnic/racial homogeneity of his monocentric case study. This feature is of fundamental importance when it comes to specific treatment since younger patients are usually more suitable for a surgical approach.

### 4.3. Which Is the Most Frequent Histology of Giant RPLs?

The most common histological type was WDLPS, which accounted for almost 50% of cases. WDLPSs are not aggressive and have a low growth rate, so they are not very symptomatic and can grow for years before being diagnosed: as the literature shows, larger tumors tend to have a lower degree of aggressiveness [141]. Despite what Gronchi et al. stated, DDLPSs can also reach considerable dimensions [141]. In fact, DDLPSs were well represented in our study (30% of cases). The reasons could depend both on their intrinsic aggressiveness and growth rate and on the fact that a WDLPS can remain inert for a period and subsequently transform into an aggressive DDLPS [2]. Regarding the other histotypes, in our review, PLPs were the least common among giant RPLs (1/157), while mixed forms were more represented than myxoids (15/157 vs. 9/157). For histological classification, we referred to the WHO classification of tumors in Soft Tissue and Bone [141].

### 4.4. How Often Is It Diagnosed Incidentally?

As previously explained, patients usually turn up to emergency departments complaining about weight gain and abdominal distension. Other symptoms reported were nausea, fainting, abdominal discomfort, and other signs associated with the mass effect like early satiety, loss of appetite, and intestinal occlusion. This behavior is common between giant and normal-size RPLs, which can grow for long periods with the same unspecific symptomatology. Unsurprisingly, the incidental diagnosis of these tumors was rare (8 on 141) and occurred only in specific situations (i.e., pregnancy).

### 4.5. How Often Is Surgery Associated with R0 Resections and How Often Is It Used for Palliation?

Some authors claim that an R0 complete resection is not always possible and, even when performed, does not completely exclude the risk of local recurrence [28,50,80,107,115,128].

Our data instead suggest that surgery was almost always performed with a curative intent. In fact, in 118/153 (77%) cases, 36/153 resections were expressly declared as R0, and in 82/153 cases, where the resection margins were not specified, we confidently assumed an R0 resection since surgery was the only treatment. Differently, in 29/153 the radicality was searched for but not found. However, in only 6/153 cases was the surgical approach used upfront as a palliative solution [20,41,75,101,102,119]. We emphasize that the sources of our data (which are all case reports) do not allow for a correct evaluation of the follow-up, although, in our cases, the therapeutic aim of surgery was frequently tried and achieved.

### 4.6. How Often Are Radio- and Chemotherapy Included in the Therapeutic Scheme?

LPSs are radiosensitive tumors, and studies have suggested that radiotherapy (RT) can reduce the risk of local recurrence and lengthen the recurrence-free interval despite its poor and controversial application [2].

When used as adjuvant therapy, RT’s main issue is that the viscera tends to fall into the resection bed, which coincides with the irradiation target, increasing the risk of adhesion and visceral toxicity. Instead, if delivered as neoadjuvant therapy, it can help to reduce the tumor dimension and help the exeresis, despite the risk of adhesive syndrome. The role of adjuvant or neoadjuvant chemotherapy remains questionable: its use is actually limited to cytoreductive neoadjuvant therapy as a means of sensitization for RT and as definitive therapy for inoperable tumors [2].

Our revision confirms that the suboptimal and controversial use of radiotherapy and chemotherapy in the treatment of normal LPSs is a relevant feature also for giant RPLs. Ostrominski et al. reported a case in which, despite neoadjuvant radiotherapy, the surgeon achieved only an R2 resection with a palliative aim [119]. Despite the cases reported by Deng et al. being treated in the last twenty years, for this section, a comparison with his study can not be performed since it excluded patients undergoing radio/chemotherapy [5]. However, there is optimism about promising radio–chemotherapeutic protocols that could change future therapeutic strategies [1].

### 4.7. What Is the Incidence of Local Recurrences and Metastasis?

RPL usually recurs within 6–48 months after the initial surgical resection with a mean tumor volume doubling time of 100 days [2]. The current guidelines from the *National Comprehensive Cancer Network* for the surveillance of soft-tissue sarcomas arising in the retroperitoneum divide the follow-up strategies between successfully resected low- and high-grade liposarcomas. Low-grade tumors should be followed up with a physical examination and total body CT scan every 3–6 months for 2–3 years and then annually. High-grade tumors undergo the same surveillance protocol for the first 2–3 years, then every 6 months for the next 2 years, and then annually [142].

The literature highlights that the only factor surely associated with local recurrence is the incomplete resection of the tumor [142]. In fact, Lewis et al. believe that, to reach a curative aim, surgeons should perform aggressive attempts to achieve a complete surgical resection, while incomplete resection should be considered only for symptom relief [4]. Mansfield et al. claim that en-bloc resection involving other organs allows surgeons to also excise non-visible microlesions and is associated with an improved recurrence rate; however, a benefit to overall survival (OS) was not demonstrated [143]. In contrast, OS improvement was shown by Deng et al. [5].

In our review, the evaluation of the relapses’ incidence on giant RPLs is eventually not reliable due to the lack of data (96/157 reports) and the inadequacy of the timings of the follow-up. In fact, there is a huge difference between what was found in our literature review (33/96 recurrences overall) and what was reported by Deng et al. with an adequate follow-up (33/41 R0 patients followed for 5 years) [5]. Further prospective studies specifically aimed to identify these data are requested to shed some light on this issue.

Regarding metastasis, we confirm what is already said in the literature: giant RPLs are tumors that tend to recur but have a low tendency to metastasize. Not surprisingly, distant metastases were more often found in the dedifferentiated histotypes.

### 4.8. What Is the Possibility of Sparing Abdominal Organs and What Are the Most Common Organs Excised Within the Tumor?

Surgery for this neoplasia has demolitive features involving several organs. The kidney is the most involved organ because of its retroperitoneal localization even if there are no reported cases of bilateral invasion. Colon and ovarian adnexa are often involved as well. These data seem to be in line with what was reported by Strauss et al. on RPS, excluding ovaries, which are rarely touched in normal-size RPSs [144]. In contrast, Deng et al. reported on 10 mono-organ and 31 multi-organ resections and found that the colon was the most resected organ, followed by the kidney [5].

Still, on the surgical side of this topic, organ sparing represents one of the toughest challenges in this field. Even if this possibility could sound attractive, it is always important to balance the risk of recurrence deriving from the persistence of a single tumoral residue in the spared organ with the quality of life deriving from organ excision itself. Although this is a key point of giant RPL surgery, in 46 of our cases, there was no express reference to organ resection, so we confidently assumed that there was no organ removal. These 46 cases, summed with the 36 in which organ exeresis was expressly not performed, show that, in half of the cases, organ sparing is a reachable objective for an expert surgeon.

## 5. Conclusions

Giant RPLs still remain an interesting challenge for surgeons. Although we cannot define giant RPLs as a completely different entity from normal-size RPLs, we can claim that they have their own characteristics: they are rare liposarcomas of the retroperitoneum which measure > 30 cm and that are not defined by their weight; sex does not seem to be a risk factor for this neoplasia; they have a significant incidence also below 50 y.o.; the most common histotypes found are WDLPSs and DDLPSs and the diagnosis is almost always symptomatic. Surgical treatment is the gold standard, even though clear resection margins do not prevent the risk of recurrence; hence, the benefits of organ exeresis to achieve R0 resections have to be balanced with the risks of the organ excision itself. For their intrinsic characteristics, these tumors have high taxes of recurrence and low rates of metastasis. Further studies are necessary to completely characterize these tumors and identify the best methods of diagnosis and therapy.

## Abbreviations

The following abbreviations are used in this manuscript:

giant RPL	giant retroperitoneal liposarcoma
Giant RPLs	giant retroperitoneal liposarcomas
DDLPS	dedifferentiated liposarcomas
PLPS	pleomorphic liposarcomas
RPS	retroperitoneal sarcomas
WDLPS	well differentiated liposarcomas
